# Timing Training in Female Soccer Players: Effects on Skilled Movement Performance and Brain Responses

**DOI:** 10.3389/fnhum.2018.00311

**Published:** 2018-08-02

**Authors:** Marius Sommer, Charlotte K. Häger, Carl Johan Boraxbekk, Louise Rönnqvist

**Affiliations:** ^1^Department of Psychology, Umeå University, Umeå, Sweden; ^2^Department of Community Medicine and Rehabilitation, Umeå University, Umeå, Sweden; ^3^Center for Demographic and Aging Research (CEDAR), Umeå University, Umeå, Sweden; ^4^Umeå Centre for Functional Brain Imaging (UFBI), Umeå University, Umeå, Sweden; ^5^Danish Research Centre for Magnetic Resonance, Centre for Functional and Diagnostic Imaging and Research, Copenhagen University Hospital, Hvidovre, Denmark

**Keywords:** timing training, neuroplasticity, fMRI, action observation, action perception, soccer

## Abstract

Although trainers and athletes consider “good timing skills” critical for optimal sport performance, little is known in regard to how sport-specific skills may benefit from timing training. Accordingly, this study investigated the effects of timing training on soccer skill performance and the associated changes in functional brain response in elite- and sub-elite female soccer players. Twenty-five players (mean age 19.5 years; active in the highest or second highest divisions in Sweden), were randomly assigned to either an experimental- or a control group. The experimental group (*n* = 12) was subjected to a 4-week program (12 sessions) of synchronized metronome training (SMT). We evaluated effects on accuracy and variability in a soccer cross-pass task. The associated brain response was captured by functional magnetic resonance imaging (fMRI) while watching videos with soccer-specific actions. SMT improved soccer cross-pass performance, with a significant increase in outcome accuracy, combined with a decrease in outcome variability. SMT further induced changes in the underlying brain response associated with observing a highly familiar soccer-specific action, denoted as decreased activation in the cerebellum post SMT. Finally, decreased cerebellar activation was associated with improved cross-pass performance and sensorimotor synchronization. These findings suggest a more efficient neural recruitment during action observation after SMT. To our knowledge, this is the first controlled study providing behavioral and neurophysiological evidence that timing training may positively influence soccer-skill, while strengthening the action-perception coupling via enhanced sensorimotor synchronization abilities, and thus influencing the underlying brain responses.

## Introduction

Skilled sports activities entail complex sequential movements, which span multiple sensory-motor effectors, and rely on neural mechanisms capable of producing well-timed and orchestrated actions in the millisecond range (see Buonomano and Laje, 2010). For instance, a precise integration between the visual and motor systems is crucial for timing the kick of a football in motion (Egan et al., 2007). Similarly, well-developed audio-motor integration is essential in order to synchronize movements with external auditory cues (sensorimotor synchronization; e.g., Repp and Su, 2013), like in contemporary dancing (Minvielle-Moncla et al., 2008).

Given that timing, i.e., sensorimotor synchronization ability, is an important component in coordinated motor actions, an improvement in this ability is expected to positively influence the precision and quality of motor skills execution, although this phenomenon is still not very elucidated. Notably, synchronized metronome training (SMT), in which persons are trained to synchronize a targeted behavior (e.g., motor coordination of upper and lower extremities) to an externally provided (auditory) target rhythm, has been found to improve the accuracy and consistency of golf shots (Libkuman et al., 2002; Sommer and Rönnqvist, 2009) and enhance performance kinematics and dynamics of the golf swing (Sommer et al., 2014). These studies indicate that improved motor timing, as an effect of SMT, is transferable to unrelated motor tasks, likely inducing unique neuro-plastic changes. This is in line with McGrew’s (2013) assumption, that SMT may result in increased neural integration and synchrony between brain regions, especially those related to more domain-general motor functions, as for instance the cerebellum and the supplementary motor areas (Meegan et al., 2000; Karmarkar and Buonomano, 2003), and the cerebellar role in time keeping (Keren-Happuch et al., 2014). Hence, as sensorimotor synchronization entails a temporal integration of perception and action (Pollok et al., 2009), improved motor timing may transfer to unrelated motor tasks through the integration of—and interaction between—sensory information from multiple modalities (e.g., Wan and Schlaug, 2010), reflecting a means for strengthening the perception-action coupling.

Numerous behavioral and brain imaging studies provide evidence for a relationship between action execution and action perception showing motor resonance effects while observing movements (e.g., Cross et al., 2013; Ménoret et al., 2013), and effects of perceptual resonance when executing actions (e.g., Cross et al., 2006; Schütz-Bosbach and Prinz, 2007). Functional brain imaging data also support the perception-action coupling, suggesting that several cortical motor regions including the inferior frontal gyrus, premotor cortex, supplemental motor area, inferior parietal lobule, as well as the cerebellum (Calvo-Merino et al., 2006; Bubic et al., 2010; Caspers et al., 2010; Christensen et al., 2014) are also activated during action observation. Perception- and action-focused research has provided important knowledge related to how sensorimotor brain regions support action recognition and understanding (Rizzolatti and Craighero, 2004) for consequent preparation of motor responses and movement imitation (Umiltà et al., 2001). Further, it has been shown that the underlying brain activation sub-serving action observation is mediated by task-specific experience (Calvo-Merino et al., 2005; Cross et al., 2006; Wright et al., 2010; Bove et al., 2017) and task-specific training (e.g., Catmur et al., 2007; Reithler et al., 2007). However, to our knowledge, no study has investigated whether brain activation sub-serving (task-specific) action observation may be susceptible to changes induced by non-task specific training.

Adaptations in brain regions involved in timing tasks are expected to have an effect on motor performance due to shared neural resources involved in motor planning and execution. Thus, it is likely that improved sensorimotor synchronization is dynamically effecting the underlying neural mechanisms. Additionally, this may (over time) create more consistency and efficiency between the cortical and sub-cortical networks and the sensory timing loops (multi-sensory integration) involved in the actual movement control/performance, as well as when simply observing the same action being performed.

The objective of the present study was therefore to investigate the effects of timing training (SMT) on a specific soccer skill and the associated changes in brain functional response in elite female players. We hypothesized that SMT would improve the players’ sensorimotor synchronization abilities, while also positively influencing soccer-kick performance by means of increased outcome accuracy. Further, we expected pre- to post-test effects of SMT to induce changes in motor cortical activity underlying passive observation of a soccer cross-pass task.

## Materials and Methods

### Participants

Twenty-five female elite soccer players participated, active in the highest or second highest soccer divisions in Sweden. The players were from three local clubs, playing in a range of outfield positions, and were all involved in regular training and match-play. Due to the notion that female soccer is underrepresented in sport-related research in general (and within soccer in particular), and also due to the effects of having a more coherent group of participants, only female soccer players were recruited for the present study. Prior to testing, the participants were randomly assigned to either an experimental SMT group (*n* = 12) or to a control group (*n* = 13). The experimental group consisted of nine players from club A, two players from club B and one player from Club C, and the control group consisted of eight players from Club A, two players from club B and three players from club C. The experimental group (aged 18.6 ± 2.4 years [M ± SD]) had an average of 12.0 ± 2.2 years of experience playing organized soccer (in a team), and the control group (aged 20.3 ± 2.9 years) an average of 12.8 ± 3.4 years of experience. There were no significant between-group differences for age or experience. All players, except one in the experimental group, reported right-foot preference for soccer skill performance.

During the period of the study both the SMT- and the Control-group continued their regular team practice (on average 6 h (four sessions) of practice a week during the 4-week intervention period). These practice sessions typically included warm-up exercises, passing-exercises, set-piece exercises (corners and free-kicks), exercises containing strategic plays and finally playing exercises (game simulation). The team-coaches stipulated these exercises to account for 70%–80% of the total practice time, and the rest of the sessions devoted to physical exercises (e.g., running). All participants signed an informed consent form prior to participation. The study was approved by the Umeå Regional Ethical Board (registration no. 2011/394-31) and conducted in accordance with the 1964 Helsinki Declaration and its later amendments or comparable ethical standards.

### Procedure

The testing took place before the competitive season started, pre-tests were held during the last week of January and the post-test during the last week of February. First, the participants’ functional brain response during observation of a soccer cross-pass task was assessed using functional magnetic resonance imaging (fMRI), initiating the data collection.

The week following the last day of fMRI pre-tests, the timing and soccer-skill pre-tests were conducted. Upon arrival at the test venue (an indoor 90 × 45 m soccer pitch with artificial turf), the players received an explanation of the experimental protocol and filled out a screening questionnaire. The participants were then subjected to the timing pre-test, followed by a 10-min standardized warm-up (running, dynamic stretching and ball handling). Finally, before assessing cross-pass performance (this cross-pass task was identical to the cross-passes observed in the fMRI paradigm), the players were allowed three practice trials with each foot to familiarize themselves with the task. The players wore the same soccer shoes at both test occasions and the same footballs were used at both test occasions for all participants (Puma Powercat 1.12: size 5; Puma Inc., Westford, MA, USA). Also, the experiment leaders were the same at both pre- and post-test, so as to eliminate inter-experimenter variability.

After the intervention period, all players performed the soccer-skill post-test, then the SMT post-test and finally the post fMRI scanning session.

### Data Acquisition

#### The Timing Skill Tests and Timing-Intervention

The Interactive Metronome^®^ (IM) system assessed the participants’ sensorimotor synchronization ability at pre- and post-test. In addition to the basic uni- and bilateral, rhythmic hand and feet movements previously described (Sommer and Rönnqvist, 2009; Sommer et al., 2014), two new tasks were added to mimic soccer related movements. One task involved kicking a wall-mounted contact-sensing trigger 30 cm above the ground, hitting the trigger with the top of the foot. The other task involved the use of four floor-mounted sensor, in which the participant was required to step on one sensor at the time in a clock-wise manner (see Supplementary Material Appendix A1). The participants’ movements are registered by contact-sensing triggers which enables the system to generate sensorimotor synchronization scores on two dependent measures; the mean millisecond discrepancy between the participant’s responses and the reference beat (timing asynchrony), and the inter-response interval that is a measure of how close each hit is timed to the previous hit (variability). The tempo of the metronome was set at 54 beats per minute (bpm), and thus with an inter onset interval (IOI) duration of 1100 ms, for all tasks. No guide sounds are available during the tests, which took about 20 min to complete.

The SMT-intervention included 12 sessions of training with The Interactive Metronome^®^ (IM) system, distributed over three 41–45 min sessions a week during a 4 weeks period after the pre-test. In short, the training invoke SMS by means of auditory pacing (entrainment), in which subjects are trained to synchronize a targeted behavior (e.g., motor coordination of upper and lower extremities) to an externally provided (auditory) rhythm heard through headphones. During training the system provides real-time auditory performance feedback entailing information about the size and direction of the asynchrony, which enables the participants to deliberately correct their timing errors as they occur. In the present study, the training regimen consisted of 13 basic uni- and bilateral exercises involving hand- and foot movements adapted from the IM system, and 10 additional exercises introducing more complex foot movements (e.g., a variety of shuffle steps and step-sequences on multiple floor-mounted sensors; see Supplementary Material Appendix A1). All exercises was performed in a standing position and the metronome reference beat was set at 54 bpm (IOI duration of 1100 ms) for most exercises (approximately in 70% of the tasks). For clarification, these movements were not related to that of kicking a ball, or other soccer specific movements. A range of different tempos (40–100 bpm) was used to maintain a challenging and ecologically sound training paradigm. Compliance was 100%; all SMT-group participants attended all training sessions. At the completion of training, participants typically engaged in approximately 27,000 motor repetitions.

#### Soccer-Kick Assessment

**Figure 1:**
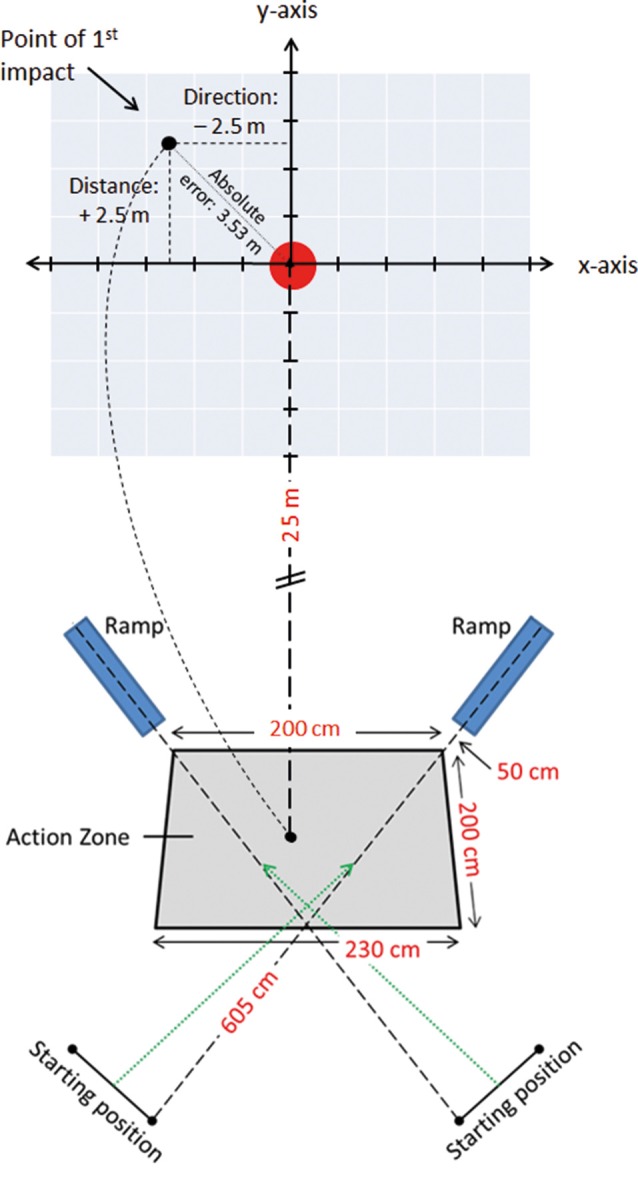
Lay-out of the 25 m cross-pass task, with a schematic of the Cartesian coordinate system and the tarpaulin sheet with the printed target, the action-zone in which the cross-pass was performed, the position of the football delivery-ramps and the players’ starting positions.

The participants were required to make a 2-m cross-pass, aiming for the center of a 5 Ø meter dart-board printed on 5 × 5 m tarpaulin sheet (Figure 1). To enhance ecological validity and mimic a ball being passed from a team-mate, the footballs were, with prior notice, randomly released from one of two ramps (with a speed of 3.8 m/s) towards a 2.0 × 2.0 × 2.3 m action zone that the player was required to make her shot within.

Participants started the tests from a standing position, 2.5 m from the action zone, and were required to jog/run into the action zone as the ball was released, thus anticipating the approach of the ball so that the shot was performed within the zone. Further, they were instructed to aim the cross-passes at the center of the floor-mounted target. No prior touches were allowed to control the ball before performing the cross-pass. The participants made 26 passes, 13 with each foot, in a counter balanced and randomized order. For balls released from the left- and right-hand ramp, passes were performed with the right and left foot, respectively. All participants engaged in a 30 s break after the first 13 trials were completed.

#### fMRI Acquisition

The fMRI data acquisition was performed on a General Electric 3.0 T scanner with a 32 channel head coil. For each functional run, a standard, whole brain, echo planar gradient-echo imaging (EPI) sequence, sensitive to blood oxygen level dependent (BOLD) contrast (T2* weighted), was used to acquire a total of 37 transverse slices (3.4 mm thickness, TR 2000 ms, TE 30 ms, flip angle = 80°, and field of view = 25 × 25 cm, 96 × 96 matrix). The first 10 scans were discarded to avoid saturation artifacts. During the fMRI scanning, each participant was lying in supine position and viewed video clips through a tilted mirror attached to the head coil. The participant wore headphones and earplugs to reduce scanner noise, and cushions placed inside the head coil were used to minimize head movement. The stimuli were presented using E-Prime 2.0 (Psychology Software Tools Inc., Sharpsburg, PA, USA).

**Figure 2:**
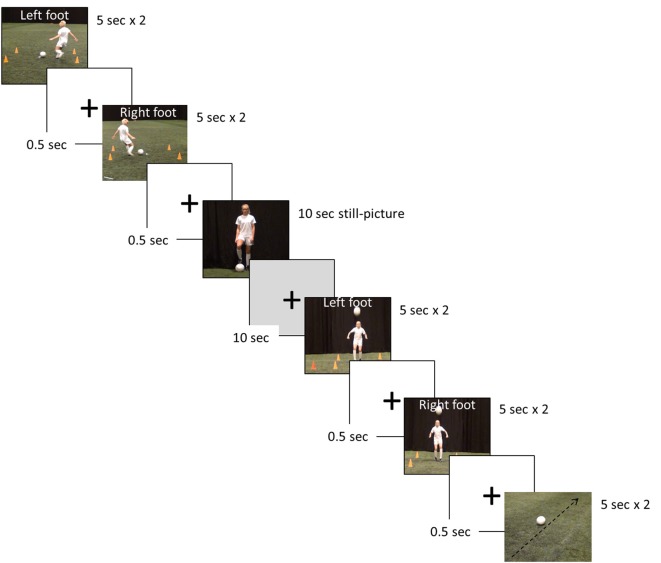
Example of the soccer skill stimuli presentation used in the functional magnetic resonance imaging (fMRI) paradigm.

The fMRI investigations assessed the hemodynamic response function (HRF) during passive observation of seven separate blocks, by use of a randomized blocked design (Figure 2). The blocks were: four digitally recorded video clips (2 × 5 s) involving two different soccer skills (performing a cross pass, and trapping a ball) performed with the left and right foot, respectively, one video clip showing a rolling football (representing a motion contrast, 2 × 5 s), one still picture of a female soccer player (10 s), and finally a black screen with a white fixation cross signifying a period of rest of 10 s.

#### Neuroimaging Analyses

All neuroimaging data was pre-processed using the following steps: realignment and unwarping, slice-timing correction, normalization to the Montreal Neurological Institute (MNI-space) template and then smoothed using a Gaussian kernel of 8 mm full-width half-maximum. The final voxel size was 2 × 2 × 2 mm. The design matrix convolved the experimental design with a HRF, and the model was estimated using a high pass filter of 128 s. Pre-processing and data analysis were completed using SPM 8 (Welcome Department of Cognitive Neurology, London, UK^1^).

The first step was to investigate the underlying functional brain response associated with (action) observation during the pre-scanning session using the cross-pass video > control video (rolling football) contrast. The general linear model was used to set up single subject analyses (first level analysis) with each of the seven blocks modeled separately. Random-effects analyses (second level analysis) were performed to reveal group-averaged data. Statistical parametric maps (SPMs) were generated using *t*-statistics. For this analysis, an FWE (*p* < 0.01) corrected statistical threshold was used. The second step was to investigate the independent effect of SMT on the underlying functional brain response associated with (action) observation of the cross-pass videos. For the second objective the pre-and post-training session were modeled together. First, we performed a flexible factorial analysis to directly examine possible group × time interactions. This analysis revealed no significant results. Therefore, we further assessed the brain regions that showed a significant difference in activation between tests (pre and post) using paired *t-test* for both groups separately. Again, we used the cross-pass video vs. control video contrast. For this analysis, we used a cluster threshold of *k* = 5 and a voxel-wise threshold, *p* < 0.005 uncorrected. Then, for each significant region the beta values of each local maxima (peak activation) from the second level analysis were plotted for each subject from both groups (BOLD plots). These values were calculated by dividing the signal change for the specific peak (cross pass > control video) of each participant with the mean signal change of the session for the same participant. In-house developed software (DataZ) was used to visualize BOLD plots. Interaction effects were subsequently calculated with repeated measures ANOVA using Statistica (StatSoft Inc., 2011; data analysis software system). Finally, we explored whether increased functional brain response was associated with improved cross-pass performance by correlating the signal change for the peaks showing a significant interaction with the change in performance for the SMT group only. To control for Type 1 error across multiple tests (correlations) a Bonferroni adjustment of the alpha-level was applied (e.g., 0.05/3 = 0.017).

#### Data Extraction and Analysis of Cross-Pass Performance

A roof-mounted SONY HDR-PJ10E (Mahwah, NJ, USA) video camera, capturing all trials at 50 frames per second, was placed 10 ms above the center of the target to record the impact of the ball on or around the tarpaulin sheet, covering a total area of 20 × 20 ms. All trials were tracked manually by assessing the point of first impact (ball-ground impact) within the visual field of the video camera array. To facilitate accurate coding of the point of first impact, a photoshop-manufactured Cartesian coordinate system was applied to each video sequence. The coordinate system consisted of 15 vertical and 15 horizontal lines with 1 m spacing between them, and with an X and a Y axis with its origin placed over the center of the target (Figure 1). Whenever the impact of the ball was between two coordinate axes, the point of impact was rounded to the closest 0.5 m. During coding, the video clip was paused when the ball arrived within the field of the video array, and then forwarded one frame (20 ms) stepwise until the point of first impact was identified.

The distance from the ball to the center of the target was measured in X (direction) and Y (distance) coordinates, with negative X-values denoting point of impact being to the left of the target, positive X-values to the right, negative Y-values short of the target, and lastly positive Y-values denoting the point of impact overshooting the target. The discrepancy in X- and Y-axis where subsequently used to calculate Absolute Error (AE) (Σ |*x_i_ − T*|/*n*), Target Variability ($\sqrt{\Sigma {\left(x_{i}-T\right)}^{2}}/n$) and Variable Error (VE) ($\sqrt{\Sigma {\left(x_{i}-M\right)}^{2}}/n$). As passing accuracy plays a major role in team play efficiency, we investigated the AE, a measure of accuracy denoting the average deviation of each participant’s total number of shots (26) from the target without respect to position. Moreover, as the spread of the 26 passes provides information about the player’s performance consistency, Target Variability, also known as root-mean-square error (TV) and VE were analyzed. TV is a measure of the participant’s total spread about the target, representing an overall measure of success in hitting the target, whereas VE denotes the variability of the participant’s deviation from his own mean (Schmidt and Lee, 1999), representing the variability or inconsistency in the accuracy of the cross-passes.

### Analysis of Soccer Skill- and Timing-Performance

Mixed within-between analyses of variance (ANOVAs) were performed with group as the independent variable, and pre- and post-test representing the within-subject repeated measure. Wilk’s lambda is reported for all interaction effects. Where the sphericity assumption was violated, Greenhouse–Geisser corrections were applied. To control for Type 1 error across multiple tests (ANOVAs) a Bonferroni adjustment of the alpha-level was applied (e.g., 0.05/3 = 0.017).

The primary consideration when deciding whether to implement an intervention or not, is if the expected change will have a practical, ecologically valid, not just a statistically significant effect on performance. Thus, in line with Hopkins (2002), we display 90% confidence limits, and give strong emphasis to the effect sizes. Effect sizes are reported as partial eta squared ($\eta_{\text{p}}^{2}$) with the following interpretation: *p* > 0.01 = small, *p* > 0.06 = medium, and *p* > 0.14 = large (Cohen, 1973).

## Results

### Effects of SMT on Sensorimotor Synchronization Ability

**Figure 3:**
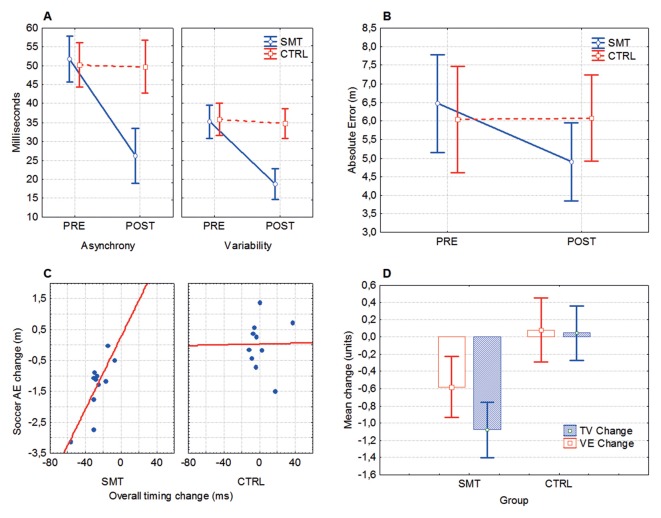
Synchronized metronome training (SMT) and cross-pass outcomes. **(A)** Mean timing asynchrony (average discrepancy from reference beat) and inter-response variability, in milliseconds. **(B)** Mean absolute error (AE) denoting the overall distance from the point of impact of the football and the target. **(C)** Changes in soccer-kick accuracy (AE, absolute error) relative to changes in motor timing performance. In both axes negative numbers denote performance improvement. **(D)** Pre- to post-test changes in target variability (TV) and variable error (VE). Box, mean + standard error (SE); whiskers, 90% confidence interval. For ANOVA outcomes, vertical bars denote 90% confidence intervals.

Timing asynchrony (mean millisecond deviation from the reference beat) and variability (inter-response interval) was significantly improved after SMT. Significant group × test interactions was evident for both timing asynchrony (*F*_(1,23)_ = 21.95, *p* < 0.001, $\eta_{\text{p}}^{2}$ = 0.49) and variability (*F*_(1,23)_ = 13.37, *p* = 0.001, $\eta_{\text{p}}^{2}$ = 0.37), indicating that pre- to post-test change in timing scores was different between groups (Figure 3A). Bonferroni corrected follow-up analysis (with an adjusted alpha value; *p* = 0.017) revealed a significant pre- to post-test improvement in timing asynchrony (*p* = 0.0001) and variability (*p* = 0.001) for the SMT group, but not for the Control group (*p*’s > 0.017). A pre- to post-test improvement (28%–79%) was seen in 13/13 of the SMT-group participants, while only 1/13 subjects in the Control group showed a test-retest change of >25% (26.9%).

### Effects of SMT on Cross-Pass Performance

For the mean absolute discrepancy from the target (AE), the ANOVA revealed a significant test × group interaction (*F*_(1,20)_ = 12.98, *p* = 0.002, $\eta_{\text{p}}^{2}$ = 0.39), indicating that SMT influence soccer cross-pass performance (Figure 3B). Bonferroni corrected follow-up analysis showed that the SMT group significantly improved the accuracy of the cross-passes from pre- (*M* = 6.46, SD = 3.22 m) to post-test (*M* = 4.89, SD = 2.34 m, *p* = 0.0003), whereas the control group did not (*p* > 0.05). A Spearman Rank Order correlation analysis revealed a significant positive correlation between (pre- to post-test) changes in timing skills and changes in cross-pass accuracy (AE) for the SMT-group (*r*_s(12)_ = 0.65, *p* = 0.03), but not for the Control group (*r*_s(12)_ = 0.10, *p* = 0.77; Figure 3C).

A multivariate ANOVA (MANOVA) showed a statistically significant between-group difference in cross-pass variability change from pre- to post-test on the combined variables TV and VE (*F*_(2,19)_ = 9.44, *p* = 0.001, $\eta_{\text{p}}^{2}$ = 0.50), indicating a greater overall improvement in outcome variability for the SMT group (Figure 3D). Univariate results from the dependent variables (utilizing a Bonferroni adjusted alpha level of 0.025) confirmed that improvement in both TV (*F*_(1,20)_ = 5.39, *p* = 0.015, $\eta_{\text{p}}^{2}$ = 0.21) and VE (*F*_(1,20)_ = 19.68, *p* = 0.0001, $\eta_{\text{p}}^{2}$ = 0.49) was significantly larger for the SMT- in comparison to the Control-group.

### Imaging Results

**Figure 4:**
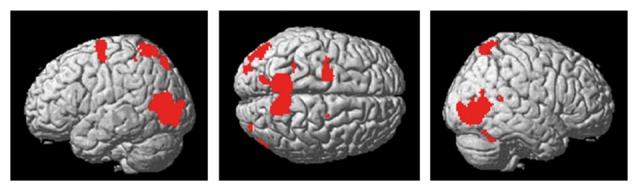
Activity in premotor-, parietal and visual regions, as well as the cerebellum, was evident during the observation of the cross-pass video relative to the control video.

The present action observation paradigm revealed activation in motor and sensory regions including the premotor cortex, parietal cortex and cerebellum, as well as in regions in the visual cortex. The anatomical location is visualized in Figure 4, and details of the activated foci are listed in Table 1 including exact location (MNI-space), *z*-score, and cluster sizes.

**Table 1:** Localization of averaged blood oxygen level dependent (BOLD) responses during observation of soccer cross-pass videos, relative to baseline, during the pre-training scan session.

		MNI coordinates				
Brain region	BA	*x*	*y*	*z*	*Z*-score	Cluster extent (*k*)
L Mid occipital gyrus (MT/V5)	37	−48	−72	10	7.94	1605
L Inferior occipital gyrus	19	−36	−84	4	7.24	
L Middle occipital gyrus	19	−36	−84	4	6.40	
L Middle occipital gyrus	18	−26	−94	10	5.58	
R Middle temporal gyrus	37	42	−62	4	6.80	1353
R Middle occipital gyrus	18	32	−88	6	5.74	
R Inferior occipital gyrus	19	38	−82	−4	5.73	
R Middle/superior temp.	39	40	−66	20	5.19	
R Mid occipital gyrus (MT/V5)	19	36	−76	12	5.08	
R Precuneus	7	10	−60	68	6.67	1664
R Superior parietal lobule	7	14	−48	50	5.91	
L Inferior parietal lobule	40	−30	−44	54	5.46	
L Superior parietal lobule	7	−18	−78	48	5.18
L Superior parietal lobule	7	−14	−76	58	5.40	
L Superior frontal gyrus	6	−20	−8	70	6.00	385
L Superior frontal gyrus	6	−26	−10	58	5.97	
L dorsal premotor cortex	6	−36	−10	62	5.47	
R Cerebellum lobule VI		36	−56	−28	5.70	85
L Cingulate gyrus	31	−18	−28	40	5.40	54
R dorsal premotor cortex	6	22	−8	60	5.27	9
R Middle temporal gyrus	22	54	−46	12	5.19	19

*Note: only activations in excess of five voxels are listed in the table. L/R, left and right hemispheres. p < 0.01 FWE corrected*.

For the independent effect of SMT on the underlying brain activation patterns associated with (action) observation of the cross-pass videos, the fMRI analysis revealed decreased responses following SMT in bilateral cerebellum, superior temporal gyrus (STG) and fusiform gyrus (FFG). See Figure 4 for visualization, and Table 2 for exact locations (MNI-space), cluster extent, and *z*-values. There were no significant changes in functional brain response between pre-and post-testing for the control group.

**Table 2:** Brain regions showing significantly decreased BOLD responses during observation of soccer cross-pass videos relative to baseline, in the intervention group, as an effect of synchronized metronome training (SMT).

		MNI coordinates				
Brain region	BA	*x*	*y*	*z*	*Z*-score	Cluster extent (*k*)
R Cerebellum lobule VI		34	−56	−28	3.18	14
R Inferior parietal lobule	40	38	−28	30	3.09	37
L Posterior cingulate gyrus	7	−14	−26	44	2.95	23
L Fusiform gyrus	37	−38	−60	−20	2.80	7
L Superior temporal gyrus	41	−48	−44	24	2.78	13
L Cerebellum		−28	−72	−18	2.65	5

*Note: the table shows transformed Z scores from an SPM{F} for the main effect of test for the SMT group. Only activations in excess of five voxels are listed in the table. L/R, left and right hemispheres. p < 0.005, uncorrected*.

**Figure 5:**
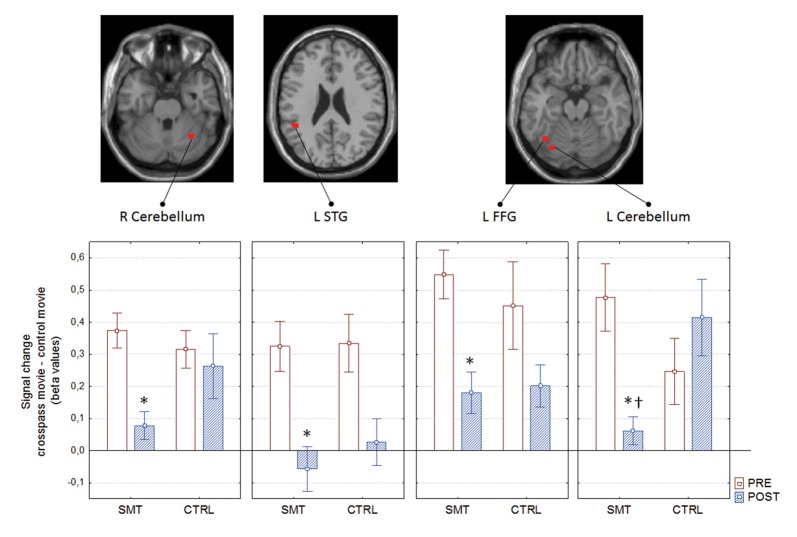
Pre- to post-test blood oxygen level dependent (BOLD) signal change as a function of group, with corresponding neural correlates. *pre- to post-test difference; ^†^between group difference; R, right; L, left; STG, superior temporal gyrus; FFG, fusiform gyrus. Boxes denotes mean and whiskers SEM.

The mixed between-within subjects ANOVA applied on the outcomes from the assessed pre- and post-test bold signal magnitude over test, groups and regions revealed a significant Group × Test interaction; *F*_(1,21)_ = 5.02, *p* = 0.035 $\eta_{\text{p}}^{2}$ = 0.19. The Bonferroni corrected follow-up analysis revealed that the only region displaying a between-group difference at post-test was the left cerebellum (*p* = 0.002). The right cerebellum displayed a close to significant difference between groups (*p* = 0.051), whereas the left STG and FFG did not prove to be significantly different between groups (Figure 5).

Decrease in right cerebellum (x: 34, y: −56, z: −28) activity during soccer kick observation showed a significant correlation with improvements in cross-pass performance (less error and variability) for the SMT group (*r*_s__(12)_ = 0.601, *p* = 0.04 for AE, and *r*_s__(12)_ = 0.671, *p* = 0.017 for target variability, respectively).

Finally, pre-to post-test activity change within the right cerebellum also showed positive correlations with pre- to post-test changes in the foot-related timing tasks for the SMT group, denoting a correspondence between decrease in neural activity and decrease in timing error (*r*_s__(12)_ = 0.657, *p* = 0.02) and inter-response intervals (*r*_s__(12)_ = 0.741, *p* = 0.005), respectively.

## Discussion

In line with our expectations, only 4 weeks of SMT improved motor timing and motor skills (by means of reduced variability and enhanced precision of soccer cross-passes) in elite- and sub-elite female soccer players, and thus corroborate previous results in golf players (Libkuman et al., 2002; Sommer and Rönnqvist, 2009; Sommer et al., 2014). Additionally, the pre- to post fMRI investigations show that SMT also seemingly induces changes in the underlying brain response associated with observing a highly familiar soccer-specific action, by means of altered cerebellar activation post SMT.

### Effects of SMT on Cross-Pass Performance

The effects of SMT on sensorimotor synchronization abilities are in line with previous SMT studies, including i.e., various groups of clinical populations (Johansson et al., 2012) and adult golfers (Libkuman et al., 2002; Sommer and Rönnqvist, 2009; Sommer et al., 2014). SMT may fine-tune the way in which the firing rates of auditory neurons entrain the firing patterns of motor neurons (Thaut, 2013), consequently leading to a closer match between motor output and rhythmic input. Importantly, improved sensorimotor synchronization was transferable to improved soccer cross-pass performance, in terms of better accuracy and less variability.

Considering that the players investigated in the present study were elite players, possible “ceiling-effects” of cross-pass performance may be expected; thus, that the players’ high level of soccer skills would not allow for evident improvements. Yet, the soccer accuracy outcomes were significantly improved and an overall, concomitant decreased variability was found.

In line with our previous findings on the effects of SMT on golf performance (Sommer and Rönnqvist, 2009; Sommer et al., 2014), although not investigated in the present study, it is plausible that SMT reinforces the coordinative structure and the temporal synchronicity of the underlying movement dynamics of the kicking motion. Our findings point toward that SMT seemingly do influence the spatio-temporal properties of a generalized motor program Schmidt and Lee (1999) for a cross-pass skill.

Alternatively, in line with the dynamic systems approach (e.g., Kelso et al., 1986), SMT may strengthen the perception-action coupling by means of a more efficient self-organization of motor performance (action), in relation to the moving ball (perception). Here, improved performance may be viewed as the players’ ability to integrate sensory information from their own body during movement (action) with relevant sensory information perceived from the environment (e.g., tau information of the football, see Lee et al., 2001). Kicking a football in motion does not only require predictive abilities related to timing the kick in accordance with the spatial and temporal constraints of the ball-flight trajectory, but also relies on the coordination of the hip, knee and ankle. However, action and perception can obviously not be separated in the example of kicking a football in motion. Thus, in order to successfully satisfy the task constraints of force and/or accuracy, the player must master the interaction of the motor and the perceptual systems.

### Effects of SMT on Brain Activation Patterns During Action Observation

Both motor representations (e.g., Olsson and Nyberg, 2011) and brain structures (Zhang et al., 2013) are assumed to be shaped by physical experience, and the amount of task-specific experience is considered to mediate brain activity patterns underlying action observation. Our findings, however, suggest that brain activity underlying action observation in one motor domain (soccer) may be influenced by training in another domain (timing training: SMT). More specifically, activation within the cerebellum, which has previously been associated with the action perception coupling (e.g., Calvo-Merino et al., 2006; Cross et al., 2013), was influenced by SMT.

It is plausible that the cerebellum is modulating the motor performance of the present cross-pass task, although we cannot provide direct evidence in our study. Our observation of altered cerebellar response following SMT, which correlated with improved cross-pass skill and sensorimotor synchronization abilities, supports cerebellum’s role in modulating motor performance. Previous research has emphasized the role of cerebellum in action observation and action recognition (e.g., Calvo-Merino et al., 2006; Cross et al., 2013; Wadden et al., 2013), suggesting that cerebellar activation is modulated by familiarity or expertise in regard to the observed action. In the present study, however, extensive training (SMT) was associated with a decrease in cerebellar lobule VI activity. Lobule VI of the cerebellum has been suggested a key structure for both perceptual and motor tasks involving timing (see meta-analysis by Keren-Happuch et al., 2014), and in the generation of internal models of motor commands (Diedrichsen et al., 2005). Moreover, past studies have reported decreased cerebellar activation within lobule VI as an effect of practice or learning (Penhune and Doyon, 2005), and indicate that a functional action perception coupling is highly related to lobule VI function (Christensen et al., 2014). Additionally, the cerebellum has been found to mediate transfer of motor skills following imagery training (Olsson et al., 2008). Hence, the present study supports previous findings that the cerebellum is heavily involved in the integration of motor information and higher-order multimodal perception (see consensus article by Baumann et al., 2015). Further, the decrease of cerebellar activation, as an effect of SMT, may reflect a streamlining of the underlying brain network communication sub-serving the action perception coupling. This is in line with recent theories on the effects of SMT on brain-based motor timing and temporal processing (McGrew, 2013), and also congruent with the neural efficient hypothesis (Haier et al., 1992) and the global efficiency hypothesis (Doyon and Benali, 2005) predicting that lower and more focused cortical activation reflects higher neural efficiency. Whether the effects of SMT taps into functions of the cerebellum sub-serving the efficiency of feed-forward and error correction processes (e.g., Diedrichsen et al., 2005; Yarrow et al., 2009; Tierney and Kraus, 2013), the formation of internal models (e.g., Miall, 2003) and/or the acquisition and integration of sensorimotor information (Thaut et al., 2008; Christensen et al., 2014) is for future studies to investigate.

In the present study, we have explored an important aspect of the perception action coupling in a small and exclusive sample of elite athletes. Still, given the promising effects of the SMT intervention, future research should attempt to replicate the present effects with larger sample sizes (but not to compromise on quality by recruiting less skilled or less experienced players), and also include male athletes and athletes from different sports, to further explore its’ generalizability and potential effects.

A larger sample of participants would also allow the use of a corrected statistical threshold in the analysis of fMRI data. Also, for future studies functional connectivity should be examined between brain regions in relation to behavioral improvements, here EEG-based measures would be of particular interest due to the superior temporal resolution compared to fMRI as was used in the present study. We acknowledge possible issues in regard to applying a correlation analysis to our small sample. Still, the correlation between change in bold signal (%) and change in cross-pass performance (as an effect of SMT) suggest that cross-pass performance is clearly associated with a decreased cerebellar activation. Importantly, due to the significant Group × Test interaction, the decreased cerebellar activity should not be considered a test-retest effect.

Also, the intervention may not provide long-lasting effects on the motor timing, soccer cross-pass performance, or the underlying brain activation in this population, which should be addressed in future investigations. Further, the body orientation of the observed player may influence neural activity in an action observation paradigm. For instance, activity within the action observation network (AON) has found to be higher when the actor faces the observer than when turned away from the observer (Kilner et al., 2006). In the present setup, the player was faced away from the observer. Thus, this may explain why our analysis did not detect activation changes (for the SMT group) in AON regions such as the ventral premotor cortex and inferior frontal gyrus.

## Conclusion

In summary, this is the first controlled study demonstrating that improved motor timing and multisensory integration, as an effect of SMT, also is associated with changes in functional brain response. The present study provides both behavioral and neurophysiological evidence that timing training positively influences soccer-skill, strengthens the action-perception coupling by means of enhanced sensorimotor synchronization abilities, and affect underlying brain responses. These findings are in accordance with the idea that SMT may result in increased brain communication efficiency and synchrony between brain regions (McGrew, 2013), which in the present study was evident by reduced activation within brain areas important for temporal planning, movement coordination and action recognition and understanding (cerebellum). Also, our results complement findings indicating that the cerebellum plays an important role in the action-perception coupling (Christensen et al., 2014), and confirm recent theories supporting a cognitive-perceptual role of the cerebellum (e.g., Roth et al., 2013). Probing the influence of timing training on the underlying brain activation during soccer specific action observation is an important approach as it provides a window into the brain plasticity associated with non-task specific (timing) training, and to the underlying brain activation of skilled performance. The present study suggests that the underlying brain activation during action observation, which is claimed to be important for action recognition and understanding (e.g., Rizzolatti and Craighero, 2004), may be influenced in other ways than through task-specific training (e.g., Calvo-Merino et al., 2005) or observational learning (e.g., Cross et al., 2013). Such knowledge of how SMT may alter brain activity within regions facilitating the action perception coupling is likely important for enhancing training techniques within sports, as well as for developing new rehabilitative techniques for many clinical populations.

## Data Availability

The raw data supporting the conclusions of this manuscript will be made available by the authors, without undue reservation, to any qualified researcher.

## Author Contributions

MS and LR: conception of the work. MS, LR, CH and CJB: design of the work, drafted the work, revised the work, final approval of the version to be published, agreement to be accountable for all aspects of the work in ensuring that questions related to the accuracy or integrity of any part of the work are appropriately investigated and resolved. MS, LR and CJB: acquisition of the data. MS and CJB: analysis and interpretation of data.

## Conflict of Interest Statement

The authors declare that the research was conducted in the absence of any commercial or financial relationships that could be construed as a potential conflict of interest.

## Footnotes

^1^https://www.fil.ion.ucl.ac.uk/spm/
