# Supplementary material for: Timing Training in Female Soccer Players: Effects on Skilled Movement Performance and Brain Responses
Source: Front Hum Neurosci. 2018 Aug 2;12:311. doi: 10.3389/fnhum.2018.00311 (PMC6082929; doi:10.3389/fnhum.2018.00311)
Supplement: Supplementary file 1 [file Data_Sheet_1.pdf]

Appendix A; Synchronized Metronome Training (SMT) paradigm

Session 1	Session 2	Session 3	Session 4	Session 5	Session 6	Session 7	Session 8	Session 9	Session 10	Session 11	Session 12
44 min	41 min	41 min	43 min	41 min	43 min	41 min	41 min	42 min	43 min	42 min	44 min
**Pre-test; Exercise 1-13, and soccer-exercise I and II**	**Exercise 1** 3 min / 54 bpm (GS) OFF	**Exercise 1** 4 min / 54 bpm	**Exercise 1** 9 min / 54 bpm	**Freestyle 1** 2 min / 78 bpm	**Exercise 1** 2 min / 54 bpm	**Exercise 10** 2 min / 54 bpm	**Exercise 1** 2 min / 54 bpm	**Exercise 5** 2 minutes	**Exercise 8** 2 min / 54 bpm	**Exercise 12** 2 minutes	**Exercise 1** 2 min / 54 bpm
**Exercise 1** 5 min / 54 bpm	**Exercise 1** 6 min / 54 bpm (GS) ON	**Exercise 4** 3 min / 54 bpm	Quick Feet 4 min / 80 bpm	Quick Feet 4 min / 90 bpm	**Exercise 2** 2 min / 54 bpm	**Exercise 11** 2 min / 54 bpm	**Exercise 2** 2 min / 54 bpm	**Exercise 6** 2 minutes	**Exercise 9** 2 min / 54 bpm	**Exercise 13** 2 minutes	**Exercise 2** 2 min / 54 bpm
**Exercise 4** 4min / 54 bpm Guidesounds off	**Exercise 2** 3 min / 54 bpm	Quick Feet 4 min / 80 bpm	**Exercise 2** 3 min / 54 bpm	Tic-Tac 3 min / 90 bpm	**Exercise 3** 2 min / 54 bpm	Quick Feet 4 min / 90 bpm	**Exercise 3** 2 min / 54 bpm	**Exercise 1** 30 min / 54 bpm	Quick Feet 4 min / 90 bpm	**Exercise 1** 38 min / 54 bpm	**Exercise 3** 2 min / 54 bpm
**Exercise 5** 2 min / 54 bpm	**Exercise 6** 3 min / 54 bpm	**Exercise 2** 3 min / 54 bpm	**Exercise 3** 3 min / 54 bpm	**Exercise 1** 19 min / 54 bpm	**Exercise 7** 2 min / 54 bpm	**Multi D Wheel** 3min / 78 bpm clockwise	**Exercise 4** 2 min / 54 bpm	**Exercise 2** 2 min / 54 bpm	**Doubl MD Wheel** 3min / 54 bpm clockwise		**Exercise 4** 2 min / 54 bpm
**Exercise 6** 2 min / 54 bpm	**Exercise 10** 4 min / 54 bpm	**Exercise 3** 3 min / 54 bpm	**Exercise 10** 3 min / 54 bpm	**On Toes** 3 min / 90 bpm	**Exercise 8** 2 min / 54 bpm	**Multi D Wheel** 3 min / 78 bpm anti clockwise	**Exercise 5** 2 min / 54 bpm	**Exercise 3** 2 min / 54 bpm	**Doubl MD Wheel** 3 min / 54 bpm anti clockwise		**Exercise 5** 2 min / 54 bpm
**Exercise 7** 3 min / 54 bpm	**Exercise 3** 3 min / 54 bpm	**Freestyle I** 2 min / 66 bpm	**Exercise 11** 3 min / 54 bpm	**Twist & Tap Left** 2 min / 54 bpm	**Exercise 9** 2 min / 54 bpm	**Shuffle step I** 3 min / 54 bpm	**Exercise 6** 2 min / 54 bpm	**Back & Forth I** 2 min / 66 bpm	**Shuffle step II** 3 min / 54 bpm		**Exercise 6** 2 min / 54 bpm
**Exercise 2** 4 min / 54 bpm	**Exercise 5** 3 min / 54 bpm	**Exercise 10** 3 min / 54 bpm	**Exercise 12** 3 min / 54 bpm	**Twist & Tap Right** 2 min / 54 bpm	**Multi D Wheel** 3min / 54 bpm (cw)	**Exercise 1** 10 min / 54 bpm	**Tic-Tac** 4 min / 100 bpm	**Back & Forth II** 2 min / 66 bpm	**Freestyle II** 2 min / 54 bpm		**Doubl MD Wheel** 3min / 78 bpm clockwise
**Exercise 3** 4 min / 54 bpm	**Exercise 11** 4 min / 54 bpm	**Exercise 11** 3 min / 54 bpm	**Tic-Tac** 3 min / 90 bpm	**Back & Forth I** 3 min / 54 bpm	**Multi D Wheel** 3min / 54 bpm (acw)	**Tic-Tac** 3 min / 100 bpm	**On Toes** 2 min / 100 bpm		**Exercise 12** 4 min / 66 bpm		**Freestyle II** 2 min / 54 bpm
**Exercise 8** 3 min / 54 bpm	**Exercise 4** 4 min / 54 bpm	**Shuffle step I** 3 min / 40 bpm Guidesounds off	**Multi D Wheel** 3 min / 66 bpm clockwise	**Back & Forth II** 3 min / 54 bpm	**Exercise 12** 3 min / 54 bpm	**On Toes** 3 min / 90 bpm	**Back & Forth I** 3 min / 66 bpm		**Tic-Tac** 3 min / 100 bpm		**Doubl MD Wheel** 3 min / 78 bpm anti clockwise
**Exercise 9** 3 min / 54 bpm	**Freestyle I** 2 min / 54 bpm	**Exercise 1** 4 min / 54 bpm	**Multi D Wheel** 3 min / 66 bpm anti clockwise		**Exercise 13** 3 min / 54 bpm	**Twist & Tap Left** 2 min / 54 bpm	**Freestyle II** 2 min / 54 bpm		**Exercise 13** 4 min / 66 bpm		**Quick Feet** 4 min / 90 bpm
	**Exercise 7** 2 min / 54 bpm	**Exercise 7** 2 min / 54 bpm	**Shuffle step I** 3 min / 46 bpm		**Exercise 4** 16 min / 54 bpm	**Twist & Tap Right** 2 min / 54 bpm	**Back & Forth II** 3 min / 66 bpm		**On Toes** 3 min / 100 bpm		**Exercise 10** 2 min / 54 bpm
	**Exercise 8** 2 min / 54 bpm	**On Toes** 3 min / 80 bpm	**Exercise 13** 3 min / 54 bpm		**On Toes** 3 min / 90 bpm	**Exercise 5** 2 min / 54 bpm	**Shuffle step II** 3 min / 40 bpm		**Back & Forth I** 3 min / 54 bpm		**Exercise 11** 2 min / 54 bpm
	**Exercise 9** 2 min / 54 bpm	**Twist & Tap Left** 2 min / 54 bpm			**Shuffle step I** 2 min / 46 bpm	**Exercise 6** 2 min / 54 bpm	**Doubl MD Wheel** 3min / 78 bpm clockwise		**Back & Forth II** 3 min / 54 bpm		**Exercise 7** 2 min / 54 bpm
		**Twist & Tap Right** 2 min / 54 bpm					**Exercise 10** 3 min / 54 bpm		**Twist & Tap Left** 2 min / 54 bpm		**Post-test; Exercise 1-13, and soccer-exercise I and II**
							**Doubl MD Wheel** 3 min / 78 bpm anti clockwise		**Twist & Tap Right** 2 min / 54 bpm		
							**Exercise 11** 3 min / 54 bpm				

**Exercise 1**	**Both hands**	Clapping hands together in a circular fashion	**Soccer exercise I**	3 floor-mats. One in front and one on each side of particpant: I _ - _I. L toeon L mat ->L toe on front mat -> R toe on front mat -> R toe on right mat -> L toe on L mat... ∞. 54 bpm
**Exercise 2**	**Right hand**	Clapping the hand towards the tigh	**Soccer exercise II**	Kicking a contact-sensing trigger placed under a beam 30 cm above the ground, hitting the trigger with the top of the foot. 54 bpm
**Exercise 3**	**Left hand**	Clapping the hand towards the tigh	**Freestyle I**	4 beats clapping hands (as in exercise 1) -> 4 beats both toes (as in exercise 4) -> 4 beats clapping hands -> 4 beats both toes... ∞
**Exercise 4**	**Both toes**	Tapping front floor-sensor, L/R toe alternately	**Freestyle II**	1 floor-mat in front of participant. 4 beats L toe -> 8 beats both thoes -> 4 beats R toe -> 4 beats both toes... ∞
**Exercise 5**	**Right toe**	Tapping front floor-sensor with R toe	**Quick Feet**	3 floor-mats. One in front and one on each side of particpant: I _ - _I. L toe L mat ->L toe front mat -> R toe front mat -> R toe right mat -> L toe on L mat... ∞
**Exercise 6**	**Left toe**	Tapping front floor-sensor with L toe	**Twist & Tap (L/R)**	3 floor-mats. One in front and one on each side of particpant: I _ - _I. Balance on one foot. Tap the three floor mats with the toe of the other foot from L-R or R-L.
**Exercise 7**	**Both heels**	Tapping rear floor-sensor, L/R heel alternately	**Multidirectional (MD) Wheel**	4 floor-mats around the participants. Starts with R toe hitting the R side of the front mat -> R toe on R mat -> R heel on R side of rear mat -> L heel on L side of rear mat -> L toe on L mat -> L toe on the L side of the front-mat -> R toe hitting the R side of the front mat... ∞
**Exercise 8**	**Right heel**	Tapping rear floor-sensor with R heel	**Double MD Wheel**	Like the Multidirectional Wheel, but now the subject is tapping the same mat twice before moving on to the next one.
**Exercise 9**	**Left heel**	Tapping rear floor-sensor with L heel	**Back & Forth I**	3 floor-mats in front of participant: --> *_-_* -->. Starting with weight on L* foot, stepping on R-L-R mat, then L foot on floor (* to iniate 3 steps (R-L-R) backward movemen... ∞
**Exercise 10**	**R Hand / L Toe**	Alternating R hand on tigh, and L toe on mat	**Back & Forth II**	As Back & Forth I, however starting with the opposite foot.
**Exercise 11**	**L Hand / R Toe**	Alternating L hand on tigh, and R toe on mat	**Tic-Tac**	Like Back and Forth, but with the addition of a wall-mounted hand sensor (+) starting the movement sequence: + *_-_*
**Exercise 12**	**Balance and tap R/L**	Balance on R foot and tap L toe on mat	**Shuffle Step**	3 floor-mats in a circular lay-out, approx. 1 m apart, and a cone in the middle. The participant moves from one mat to the next utilizing shuffle-steps (sideways skipping)
**Exercise 13**	**Balance and tap L/R**	Balance on L foot and tap R toe on mat	**On Toes**	1 floor-mat in front of the participant. Utilizing a skipping-movement to alternate tapping with L/R toe.
